# Solution of Iodo-Bromide Calcium Compound

**Published:** 1875-09

**Authors:** 


Solution of Iodo-Bromide of Calcium Compound.—The following are the results of a chemical analysis we have recently made of this preparation. One fluid ounce contains approximately:

| | | |
|---|---|---|
| Chloride of calcium (anhydrous) . . . | 142 | grains. |
| Chloride of magnesium, . . . . . . . | 30 | grains. |
| Chloride of sodium, . . . . . . . . | 18 | grains. |
| Bromide of magnesium, . . . . . . . | 6 | grains. |
| Iodide of potassium, . . . . . . . . | 1½ | grains. |
| Total mineral constituents, . . . . . | 200 | grains. |

—*Detroit Review.*

The Springfield (Mass.) *Republican* gives us the item that a boy child of Dr. Shea, born last month, was weighed immediately after its birth, by Dr. Rice, in the presence of a dozen witnesses, with balance scales, and weighed twenty pounds and two ounces. Can this be beat?

